# The learning curves of major laparoscopic and robotic procedures in urology: a systematic review

**DOI:** 10.1097/JS9.0000000000000345

**Published:** 2023-05-03

**Authors:** Baldev Chahal, Abdullatif Aydin, Mohammad S.A. Amin, Azhar Khan, Muhammad S. Khan, Kamran Ahmed, Prokar Dasgupta

**Affiliations:** a MRC Centre for Transplantation, Guy’s Hospital, King’s College London; b Department of Urology, King’s College Hospital NHS Foundation Trust; c Urology Centre, Guy’s and St. Thomas’ NHS Foundation Trust, London; d Oxford University Clinical Academic Graduate School, Oxford, UK

**Keywords:** laparoscopic, learning curves, review, robotic, urology

## Abstract

Urology has been at the forefront of adopting laparoscopic and robot-assisted techniques to improve patient outcomes. This systematic review aimed to examine the literature relating to the learning curves of major urological robotic and laparoscopic procedures.

**Methods:**

In accordance with PRISMA (Preferred Reporting Items for Systematic Reviews and Meta-Analyses) guidelines, a systematic literature search strategy was employed across PubMed, EMBASE, and the Cochrane Library from inception to December 2021, alongside a search of the grey literature. Two independent reviewers completed the article screening and data extraction stages using the Newcastle–Ottawa Scale as a quality assessment tool. The review was reported in accordance with AMSTAR (A MeaSurement Tool to Assess systematic Reviews) guidelines.

**Results:**

Of 3702 records identified, 97 eligible studies were included for narrative synthesis. Learning curves are mapped using an array of measurements including operative time (OT), estimated blood loss, complication rates as well as procedure-specific outcomes, with OT being the most commonly used metric by eligible studies. The learning curve for OT was identified as 10–250 cases for robot-assisted laparoscopic prostatectomy and 40–250 for laparoscopic radical prostatectomy. The robot-assisted partial nephrectomy learning curve for warm ischaemia time is 4–150 cases. No high-quality studies evaluating the learning curve for laparoscopic radical cystectomy and for robotic and laparoscopic retroperitoneal lymph node dissection were identified.

**Conclusion:**

There was considerable variation in the definitions of outcome measures and performance thresholds, with poor reporting of potential confounders. Future studies should use multiple surgeons and large sample sizes of cases to identify the currently undefined learning curves for robotic and laparoscopic urological procedures.

## Introduction

Highlights

- The robot-assisted prostatectomy learning curve ranges from 10 to 250 cases.
- The robot-assisted partial nephrectomy curve ranges from 4 to 150 cases.
- Retroperitoneal lymph node dissection and laparoscopic cystectomy learning curves are undefined.
- Standardized reporting of outcomes is required to enable future meta-analysis.

The concept of a ‘learning curve’ was first described in the aeronautics industry in 1936 to illustrate the correlation between improvements in the production of plane components and the increasing experience of the workforce involved^1^. It has subsequently been adopted in the context of surgical practice, where it has been defined as the number of cases that a surgical trainee needs to undertake in order to reach the point where their inexperience no longer affects the outcomes of the procedure^2^. This particular stage in their acquisition of skills is represented on conventional learning curve graphs by a plateau^3^. Notably, although learning curves map the achievement of technical skills proficiency, surgeons must also achieve proficiency in non-technical skills in order to gain overall surgical competency in the procedure^4^.

Various learning curve metrics are used to plot the surgical learning curve. Operative time (OT) and estimated blood loss (EBL) are two of the most commonly reported learning curve metrics^5^, with reductions in these variables being associated with surgical training progression^6^. Urology-specific patient-outcome variables include measures of potency and continence postoperatively, the impairment of which can have a significant impact on the patient’s quality of life^7^. One or more of four principal methods of analysis are then used to assess change in performance as case number increases^8^; graphical inspection, the split-group method (dividing the data into consecutive groups and comparing group means), logistic regression and cumulative sum (CUSUM) analysis.

It is highly important to define the learning curve for surgical procedures because detrimental outcomes associated with surgeon inexperience can have a major impact on patient safety, as exemplified by findings of the Bristol Royal Infirmary enquiry^9^. Knowledge of the learning curve enables the safeguarding of patients by identifying trainees in the learning phase and providing them with adequate supportive measures such as close senior supervision and additional simulation-based skill practice^10^. Mapping the learning curve can also inform the design of surgical training programmes by illustrating the impact of educational interventions on the learning process. This is particularly relevant in the context of the laparoscopic and robotic approaches which have been widely adopted as the standard of care for urological operations such as prostatectomy and radical nephrectomy. Given their relatively recent implementation in contrast to traditional open approaches, knowledge of their learning curves is important in informing and improving training programmes for these modalities so as to effectively achieve competency across all outcome measures and ensure patient safety.

The last systematic review to include an evaluation of the learning curve for laparoscopic prostatectomy was conducted in 2014^11^. The most recent systematic review evaluating the learning curve for robot-assisted laparoscopic prostatectomy (RALP), robot-assisted radical cystectomy (RARC), robot-assisted partial nephrectomy (RAPN) and robotic pyeloplasty, conducted database searching in February 2018^12^, although this particular review excluded studies published prior to January 2012 as well as single-surgeon studies which constitute the bulk of the relevant learning curve literature base.

The principal aim of this review is to provide an updated insight into the learning curves for major urological robotic and laparoscopic procedures to act as a reference for expected progress. Other aims include the evaluation of the methods used to analyse the learning curves and to provide recommendations for future studies in the field in terms of their scope and methodology.

## Methods

### Design

A systematic review was performed utilising the Preferred Reporting Items for Systematic Reviews and Meta-Analyses (PRISMA), Supplemental Digital Content 1, http://links.lww.com/JS9/A423 statement^13^ and was prospectively registered on the International Prospective Register of Systematic Reviews (PROSPERO) database (ID number: CRD42021251186)^14^. The review was reported in line with the AMSTAR-2 (A MeaSurement Tool to Assess systematic Reviews) checklist^15^, Supplemental Digital Content 2, http://links.lww.com/JS9/A424, achieving a moderate quality score.

### Eligibility criteria

The participants for which the learning curve was mapped were surgeons at any stage of training with no restriction placed on their prior surgical experience. Studies conducted in a simulated surgical setting were excluded. The interventions included were RALP, laparoscopic radical prostatectomy (LRP), RAPN, laparoscopic nephrectomy (including both conventional laparoscopic and hand-assisted techniques), RARC, laparoscopic radical cystectomy (LRC), robotic adult and paediatric pyeloplasty, laparoscopic adult and paediatric pyeloplasty, robotic retroperitoneal lymph node dissection (RPLND) and laparoscopic RPLND. Studies that reported outcomes for multiple eligible procedures but failed to provide separate data for each procedure were excluded.

Randomised controlled trials, non-randomised interventional studies, cohort studies, case series and case–control studies evaluating the learning curve were included. No language restriction was placed on studies. Review articles, conference abstracts, editorials and letters were excluded.

### Search strategy

A systematic literature search strategy was employed across PubMed, EMBASE and the Cochrane Library from inception to December 2021 for eligible studies. Both free-text terms and medical subject headings (MeSH) were used in the database search strategies as listed in the Appendix, Supplemental Digital Content 3, http://links.lww.com/JS9/A425. To find relevant full-text articles in the ‘grey literature’, the Google Scholar search engine was used alongside website searching and citation chaining.

### Study selection and screening

Two independent reviewers performed the initial article title and abstract screening and then screened the full text of the potentially eligible articles against the inclusion criteria. Where discrepancies were encountered, a third reviewer was consulted to resolve the disagreement.

### Data extraction and risk of bias assessment

The two independent reviewers used a pre-defined, standardised form to undertake the data extraction. No specific risk of bias assessment tool exists for learning curve studies, so the two reviewers utilised the Newcastle–Ottawa scale^16^ as a quality assessment tool. As before, disagreements between the reviewers over the risk of bias scores for the included studies were resolved by referral to a third reviewer.

### Data synthesis

It was not possible to perform a meta-analysis of the data collected due to substantial heterogeneity in the included studies, so the results were narratively synthesised in accordance with PRISMA^13^ guidelines, Supplemental Digital Content 1, http://links.lww.com/JS9/A423, with summary diagrams produced to provide the range of values for which the learning curve associated with a particular procedure and its outcomes were numerically defined. This provides surgeons at any stage of training with a guide of expected progress against which they can compare their own performances. Similar descriptive and diagrammatic approaches have been utilised in previous systematic reviews evaluating urological learning curves^11,17^, where meta-analysis was not possible.

## Results

**Figure 1:**
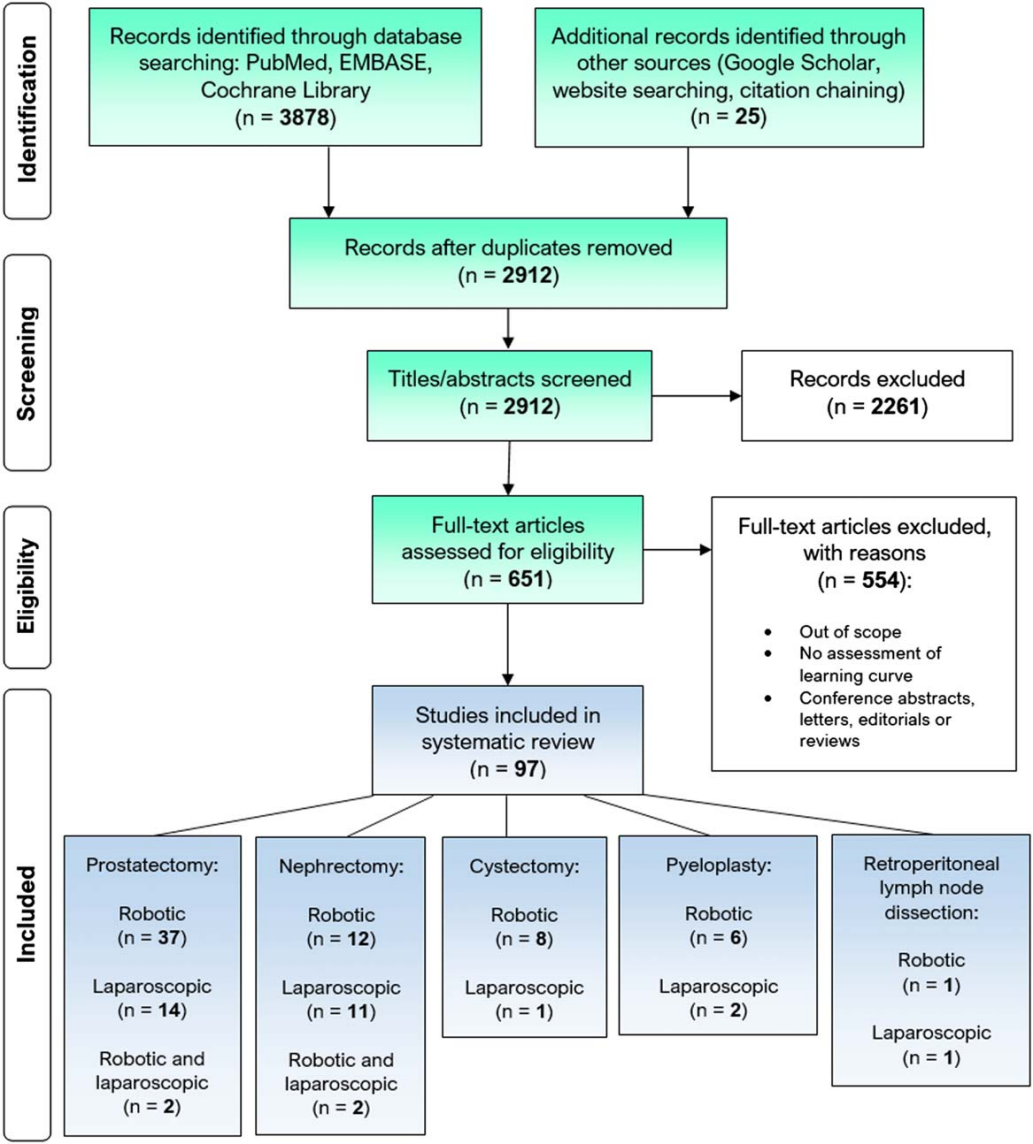
PRISMA (Preferred Reporting Items for Systematic Reviews and Meta-Analyses) diagram.

Three thousand eight hundred and seventy-eight records were identified through database searching, with an additional 25 records identified through a search of the grey literature and citation chaining. After deduplication, 2912 studies were eligible for the title and abstract screening. Two thousand two hundred and sixty-one studies were then excluded, so 651 studies underwent full-text screening. Five hundred and fifty-four studies were then excluded, with 97 studies thus included for narrative synthesis in this review. Figure 1 illustrates this study’s selection and screening process.

### Characteristics of included studies

Fifty-one studies were retrospective in design whilst 46 were prospective studies. Fifty-five studies mapped the learning curve of single surgeons, 39 involved multiple surgeons (with one study^18^ including as many as 744 surgeons) and 3 did not report the exact number of surgeons involved. The number of procedures ranged from 20^19^ to 27 348^18^ with study durations ranging from 10 months^20^ to 259 months^21^. The earliest publication date was 2000^22^, with the most recent included study published in April 2021^23^.

Table 1 displays the full study characteristics of the included studies.

**Table 1:** Characteristics of the included studies.

Study	Procedure, country	Study design	Number of surgeons	Previous experience	Number of procedures	Study duration (months)
Adili *et al*. (2017)^24^	RALP (transperitoneal), Canada	Prospective	1	600 LRPs	400	37
Ahlering *et al*. (2003)^20^	RALP (extraperitoneal), USA	Prospective	1	Experienced in open	45	10
Alemozaffar *et al*. (2012)^25^	RALP, USA	Retrospective	1	76 open RPs, 11 months’ fellowship experience of RALP (without nerve-sparing)	400	20
Al-Hathal and El-Hakim (2013)^26^	RALP, Canada	Prospective	1	NR	250	72
Atug *et al*. (2006)^27^	RALP, USA	Retrospective	3	Experienced in open and laparoscopic	100	21
Bravi *et al*. (2019)^28^	RALP (transperitoneal), Italy	Retrospective	9	8 with no experience in robotics1 with >50 RALP experience	1827	132
Chan and Pautler (2019)^29^	RALP, Canada	Retrospective	1	Fellowship-trained	577	65
Chang *et al*. (2016)^30^	RALP (transperitoneal), China	Retrospective	3	1 experienced in open only1 experienced in laparoscopic only1 experienced in open and laparoscopic	388	41
Chen *et al*. (2020)^31^	RALP (transperitoneal), China	Prospective	1	Experienced in ORP, no LRP experience	500	67
Davis *et al*. (2014)^18^	RALP, USA	Retrospective	744	NR	27348	72
Dev *et al*. (2012)^32^	RALP (extraperitoneal), UK	Prospective	1	Experienced in laparoscopic	150	NR
Di Pierro *et al*. (2015)^33^	RALP (with ePLND), Italy	Prospective	1	Experienced in open and laparoscopic	233	47
Doumerc *et al*. (2010)^34^	RALP (transperitoneal), Australia	Prospective	1	Experienced in open	212	35
Fossati *et al*. (2017)^35^	RALP, Italy	Prospective	4	NR	1477	96
Galfano *et al*. (2021)^36^	RALP (Retzius-sparing), 12 international centres	Retrospective	14	12 experts at standard RALP2 had no first-hand RALP experience	626	84
Good *et al*. (2015)^37^	RALP (transperitoneal), UK	Retrospective	1	Experienced in open and laparoscopic RPs	531	73
	LRP, UK	Retrospective	2	1 with 19 open RP and 5 LRP fellowship experience1 with 160 LN, 20 open RP experience	550	104
Gumus *et al*. (2011)^38^	RALP (extraperitoneal), Turkey	Prospective	1	No laparoscopic experience	120	27
Hashimoto *et al*. (2013)^39^	RALP, Japan	Prospective	1	500 open RPs, no laparoscopic experience	200	60
Herrell and Smith (2005)^40^	RALP, USA	Prospective	1	Highly experienced in open	350	NR
Islamoglu *et al*. (2018)^41^	RALP (transperitoneal), Turkey	Retrospective	1	Experienced in open and laparoscopic RPs	111	31
Ko *et al*. (2009)^42^	RALP (transperitoneal), South Korea	Prospective	1	>300 open RPs and >20 laparoscopic RPs	63	13
Lee *et al*. (2010)^43^	RALP (transperitoneal), South Korea	Prospective	1	NR	307	35
Monnerat Lott *et al*. (2018)^45^	RALP (transperitoneal), Brazil	Prospective	2	10 years’ experience of open RP	119	37
Maddox *et al*. (2013)^44^	RALP (extraperitoneal), USA	Prospective	3	1 with limited experience with laparoscopic RP1 with significant exposure to RALP1 with no robotic experience	575	49
Ohwaki *et al*. (2020)^46^	RALP, Japan	Retrospective	4	NR	540	76
Ou *et al*. (2011)^47^	RALP, Taiwan	Prospective	1	Open and laparoscopic experience	200	49
Pardalidis *et al*. (2008)^48^	RALP (transperitoneal), Greece	Prospective	1	NR	40	8
Patel *et al*. (2005)^49^	RALP, USA	Prospective	1	Experienced in open	200	18
Ploussard *et al*. (2010)^50^	RALP (extraperitoneal), France	Prospective	2	Experienced in LRP	206	89
Samadi *et al*. (2007)^51^	RALP, USA	Prospective	1	Experienced in laparoscopic	70	22
Sammon *et al*. (2010)^52^	RALP (extraperitoneal), USA	Prospective	3	2 experienced in laparoscopic, 1 not	225	36
Sharma *et al*. (2011)^53^	RALP (extraperitoneal), UK	Prospective	2	1 experienced in open prostatectomy1 experienced in laparoscopy	500	48
Sivaraman *et al*. (2017)^54^	RALP, France	Retrospective	9	250 LRP experiences each	1701	180
	LRP, France	Retrospective	9	NR	3846	180
Slusarenco *et al*. (2020)^55^	RALP (transperitoneal), Russia	Retrospective	1	Experienced in laparoscopy	145	24
Song *et al*. (2020)^56^	RALP (transperitoneal), South Korea	Retrospective	1	No experience of any form of RP	480	59
Thompson *et al*. (2018)^57^	RALP, Australia	Prospective	1	>3000 ORPs	1520	120
van der Poel *et al*. (2012)^58^	RALP, Netherlands	Prospective	2	>50 ORPs each	440	60
Williams *et al*. (2011)^59^	RALP (transperitoneal), Canada	Prospective	1	>200 open and laparoscopic RPs	158	53
Wu *et al*. (2008)^60^	RALP (extraperitoneal), Taiwan	Prospective	1	Experienced in laparoscopic (limited RP experience)	24	25
Baumert *et al*. (2004)^61^	LRP (transperitoneal), France	Retrospective	1	Performed >30 various laparoscopic procedures, assisted 20 LRPs	100	16
Di Gioia *et al*. (2013)^62^	LRP (transperitoneal), Brazil	Retrospective	1	Experienced in open prostatectomy and upper tract laparoscopy	240	84
Dias *et al*. (2017)^63^	LRP (transperitoneal), Brazil	Prospective	2	RALP experienced, trained in LRP	110	20
Eden *et al*. (2009)^64^	LRP (transperitoneal initially, then extraperitoneal), UK	Prospective	1	Experienced in open RP and reconstructive laparoscopy	1000	93
Eden *et al*. (2013)^65^	LRP (transperitoneal, ePLND)	Retrospective	1	Open RP, RALP, performed or supervised 868 LRPs (8 ePLNDs)	500	48
Handmer *et al*. (2018)^66^	LRP, Australia	Retrospective	9	Fellowship-trained in LRP	2943	132
Hruza *et al*. (2010)^67^	LRP (transperitoneal initially, then extraperitoneal), Germany	Prospective	5	1 first-gen surgeon (only open exp.)2 second-gen surgeons (open exp., trained by first-gen)2 third-gen surgeons (limited open exp., trained by first-gen or second-gen)	2200	96
Mason *et al*. (2016)^68^	LRP (extraperitoneal), UK	Prospective	2	Experienced in laparoscopic	500	102
Mitre *et al*. (2013)^69^	LRP (extraperitoneal), Brazil	Prospective	1	Experienced in other laparoscopic procedures	165	94
Poulakis *et al*. (2005)^70^	LRP (transperitoneal initially, then extraperitoneal), Germany	Prospective	1	>100 open RPs, no laparoscopic experience	50	17
Rodriguez *et al*. (2010)^71^	LRP (extraperitoneal), USA	Retrospective	1	NR	400	30
Secin *et al*. (2010)^72^	LRP, international	Retrospective	51	1 with 285 LRP experience50 with open RP experience	8544	102
So *et al*. (2011)^73^	LRP (mostly transperitoneal, last 11 were extraperitoneal), South Korea	Retrospective	1	Fellowship-trained in laparoscopy	100	31
Vickers *et al*. (2009)^74^	LRP, European and North American institutions	Retrospective	29	Only 1 with LRP experience	5328	113
Bajalia *et al*. (2020)^75^	RAPN, USA	Retrospective	1	Fellowship-trained	418	135
Castilho *et al*. (2020)^76^	RAPN, Brazil	Retrospective	1	No laparoscopic experience	101	48
Dias *et al*. (2018)^77^	RAPN, India	Prospective	1	Experienced in laparoscopy	108	59
Hanzly *et al*. (2015)^78^	RAPN, USA	Retrospective	1	RALP experience, no LPN experience	116	NR
	LPN, USA	Retrospective	2	Fellowship experience	116	NR
Haseebuddin *et al*. (2010)^79^	RAPN, USA	Prospective	1	>200 LPNs	38	12
Larcher *et al*. (2019)^80^	RAPN, 2 European centres	Prospective	Multiple	NR	457	132
Lavery *et al*. (2011)^81^	RAPN, USA	Retrospective	1	>100 LPNs, 100 RALPs, 15 robotic pyeloplasties	20	21
Motoyama *et al*. (2020)^82^	RAPN, Japan	Retrospective	1	300 RALPs, >100 open PNs, 15 LPNs	65	20
Mottrie *et al*. (2010)^83^	RAPN, Belgium	Prospective	1	15 LPN, experienced in robotic	62	39
Omidele *et al*. (2018)^84^	RAPN, USA	Retrospective	1	Fellowship-trained in robotics and laparoscopy	131	91
Pierorazio *et al*. (2011)^85^	RAPN, USA	Retrospective	1	Experienced in minimally invasive surgery	48	NR
	LPN, USA	Retrospective	1	Experienced in minimally invasive surgery	102	NR
Tobis *et al*. (2012)^86^	RAPN, USA	Retrospective	3	Experienced in minimally invasive surgery	100	30
Xie *et al*. (2016)^87^	RAPN, China	Retrospective	1	>1000 LPNs	144	12
Zeuschner *et al*. (2021)^88^	RAPN, Germany	Retrospective	7	NR	500	132
Azawi *et al*. (2014)^89^	HALPN (with early arterial clamp removal), Denmark	Prospective	3	NR	60	21
Bhayani (2008)^90^	LPN, USA	Retrospective	1	Fellowship-trained in LPN	50	30
Gaston *et al*. (2004)^19^	HALN, USA	Prospective	6	Residents each with experience of ≥15 open nephrectomies	30	22
Gill *et al*. (2001)^91^	LRN, USA	Prospective	1	NR	100	35
Gozen *et al*. (2017)^21^	LRN, Germany	Prospective	6	1st gen surgeons: experienced in open only.2nd gen surgeons: experienced in open, trained by 1st gen.3rd gen surgeons: no open experience, trained by 1st or 2nd gen	330	259
Jeon *et al*. (2009)^92^	LRN, South Korea	Retrospective	3	No laparoscopic experience	150	59
Kanno *et al*. (2006)^93^	LN, Japan	Retrospective	6	NR	78	47
Kawauchi *et al*. (2005)^94^	HALN, Japan	Retrospective	18	Each performed ≥20 open RNs or nephroureterectomies	166	NR
Link *et al*. (2005)^95^	LPN, USA	Retrospective	1	Experienced in laparoscopic	178	63
Masoud *et al*. (2020)^96^	LN, Egypt	Prospective	1	Trainee urologist, access to laparoscopic simulation training	40	28
Porpiglia *et al*. (2013)^97^	LPN, Italy	Prospective	1	NR	302	132
Collins *et al*. (2014)^98^	RARC (with intracorporeal neobladder), Sweden	Prospective	2	NR	67	106
Dell’Oglio *et al*. (2020)^99^	RARC (with intracorporeal urinary diversion), Belgium	Prospective	2	Limited robotic experience (<20 cases)	164	156
Guru *et al*. (2009)^100^	RARC (with ePLND), USA	Retrospective	1	Experienced in RALP, formal robotic fellowship	100	34
Hayn *et al*. (2011)^101^	RARC, USA	Prospective	1	Experienced in robotics	164	45
Hellenthal *et al*. (2010)^102^	RARC, 15 international centres	Prospective	22	NR	513	72
Honore *et al*. (2019)^103^	RARC, Australia	Retrospective	1	Experienced robotic pelvic surgeon	100	72
Pruthi *et al*. (2008)^104^	RARC (with extracorporeal urinary diversion, USA	Retrospective	1	Experienced in open cystectomy and RALP	50	23
Wang *et al*. (2019)^105^	RARC (with intracorporeal urinary diversion), USA	Retrospective	2	Fellowship-trained	56	39
						
Aboumarzouk *et al*. (2012)^106^	Laparoscopic radical cystectomy (with PLND and urinary diversion), Poland	Prospective	1	NR	65	61
Chammas *et al*. (2019)^107^	Robotic pyeloplasty (adult), Brazil	Prospective	1	Experienced in open	100	62
Cundy *et al*. (2015)^108^	Robotic pyeloplasty (paediatric), UK	Prospective	1	Experienced in laparoscopy	90	92
Dothan *et al*. (2021)^109^	Robotic pyeloplasty (paediatric), Israel	Retrospective	1	Experienced in open and laparoscopic pyeloplasty	33	192
Kassite *et al*. (2018)^110^	Robotic pyeloplasty (paediatric), France	Retrospective	2	Experienced in laparoscopy (but no laparoscopic pyeloplasty experience)	42	121
Sorensen *et al*. (2011)^111^	Robotic pyeloplasty (paediatric), USA	Retrospective	2	Experienced in laparoscopy (but minimal laparoscopic pyeloplasty experience)	33	34
Tasian *et al*. (2013)^112^	Robotic pyeloplasty (paediatric), USA	Prospective	5	4 fellows with adult robotic experience1 attending with paediatric robotic experience	100	48
Calvert *et al*. (2008)^113^	Laparoscopic pyeloplasty (adult), UK	Retrospective	NR	NR	49	60
Panek *et al*. (2020)^114^	Laparoscopic pyeloplasty (paediatric), Poland	Retrospective	1	NR	95	120
Janetschek *et al*. (2000)^22^	Laparoscopic RPLND, Austria	Retrospective	NR	NR	64	76
Schermerhorn *et al*. (2021)^23^	Robotic RPLND, USA	Retrospective	4	Trained in robotics and laparoscopy	121	120

ePLND, extended pelvic lymph node dissection; gen., generation; HALN, hand-assisted laparoscopic nephrectomy; HALPN, hand-assisted laparoscopic partial nephrectomy; LN, lymph nodes; LPN, laparoscopic partial nephrectomy; LRN, laparoscopic radical nephrectomy; LRP, laparoscopic radical prostatectomy; NR, not reported; ORP, open radical prostatectomy; PLND, pelvic lymph node dissection; PN, partial nephrectomy; RALP, robot-assisted laparoscopic prostatectomy; RAPN, robot-assisted partial nephrectomy; RARC, robot-assisted radical cystectomy; RN, radical nephrectomy; RP, radical prostatectomy; RPLND, retroperitoneal lymph node dissection.

### Risk of bias assessment

Included studies scored either 5 or 6 on the Newcastle–Ottawa scale, scoring lowest in the ‘Selection’ domain due to the potential for selection bias and the lack of control groups.

### Findings

The main findings of the included studies are listed in Table 2. Where studies defined the learning curve for ‘overall performance’, this referred to the number of cases required to achieve competency across the range of outcomes they measured.

**Table 2:** Results of the included studies.

Study	Procedure, country	Outcome measures	Learning Curve Model, statistical analysis	Thresholds in surgeon performance	Learning curve, outcome measure: number of cases
Adili *et al*. (2017)^24^	RALP (transperitoneal), Canada	PSM, OT, EBL, LOS	Split-group (quartiles); logistic regression model, Student’s *t*-test	Performance in most recent quartile used as reference standard	PSM: no statistically significant learning curve, OT, EBL: reductions from first quartile compared to last quartile
Ahlering *et al*. (2003)^20^	RALP (extraperitoneal), USA	OT, individual step time, EBL, LOS, PSM, C, TR, post-op Hb decrease, continence, potency	Graphical inspection, split-group; no tests for statistical significance reported	Number of cases to achieve 4 h proficiency	OT: 12
Alemozaffar *et al*. (2012)^25^	RALP, USA	PSM, EPIC sexual function (at 5 and 12 months), potency (at 5 and 12 months)	Split-group (octiles); linear regression models, Cochran–Armitage trend test	Number of cases to reach plateau	EPIC sexual function: 250–300
Al-Hathal and El-Hakim (2013)^26^	RALP, Canada	OT, EBL, C, TR, CR, LOS, PSM, continence, potency	Split-group (quintiles); no tests for statistical significance reported	Number of cases to reach plateau	OT: 50, PSM (pT2): 50
Atug *et al*. (2006)^27^	RALP, USA	PSM	Split-group (thirds); *χ* ^2^ test, ANOVA	Reduction in PSM rates	PSM: ≈30
Bravi *et al*. (2019)^28^	RALP (transperitoneal), Italy	PSM, BCR	Multivariable logistic regression model; Wilcoxon rank-sum test, *χ* ^2^ test, multivariable Cox regression model	Number of cases to reach plateau	PSM: 200
Chan and Pautler (2019)^29^	RALP, Canada	C, continence, LOS, BCR	CUSUM method; no tests for statistical significance reported	Number of cases to reach plateau	C: ≈500
Chang *et al*. (2016)^30^	RALP (transperitoneal), China	OT, LOS, EBL, PSM, continence, BCR	Graphical inspection, split-group (groups of 20); *χ* ^2^ test, Student–Newman–Keuls test, ANOVA, Kruskal–Wallis test	Number of cases to reach plateau	For open exp surgeon: OT and EBL plateau after 20No plateau for lap-exp surgeonFor surgeon exp. in both open and lap: OT plateaus after 40, no plateau for EBL
Chen *et al*. (2020)^31^	RALP (transperitoneal), China	OT, EBL, LOS, PSM	Split-group (quintiles); ANOVA, Kruskal–Wallis test	Number of cases to reach plateau	OT, EBL, LOS: 200
PSM: no statistically significant learning curve					
Davis *et al*. (2014)^18^	RALP, USA	OT, LOS, C, CR	Split-group; Student’s *t*-test, *χ* ^2^ test, Jonckheere–Terpstra test	NR	OT, LOS and C all improve in first 100 with additional improvements in next 100
Dev *et al*. (2012)^32^	RALP (extraperitoneal), UK	OT, individual step time, EBL, LOS, PSM	Split-group; linear regression model, Wald test	NR	Individual step times all significantly decreased between the first 25 and last 50 procedures, except for the Rocco stitch step
Di Pierro *et al*. (2015)^33^	RALP (with ePLND), Italy	OT, time for ePLND, C, resected lymph node yield, positive lymph nodes, PSM	Split-group (quartiles); *χ* ^2^ test, Kruskal–Wallis test, logistic regression models, mixed linear regression models	Number of cases to reach plateau	Lymph node yield: 60
Doumerc *et al*. (2010)^34^	RALP (transperitoneal), Australia	OT, EBL, TR, C, LOS, PSM, continence	Join point regression model; Monte Carlo permutation method, *χ* ^2^ test, ANOVA	Number of cases to reach 3 h proficiencyNumber of cases to reach plateauNumber of cases to match open performances	OT: 110 (3 h proficiency)PSM (pT2): plateau at 140PSM (pT3): plateau at 170200 for early continence (6 weeks)
Fossati *et al*. (2017)^35^	RALP, Italy	Continence	Graphical inspection; multivariable Cox regression analysis	NR	Continence: no plateau reached after >200>400 to increase continence recovery rate at 1 year from 60% to ≈90%
Galfano *et al*. (2021)^36^	RALP (Retzius-sparing), 12 international centres	OT, console time, EBL, TR, C, LOS, continence, potency, PSM, BCR	Split-group (halves), graphical inspection with LOWESS; Mann–Whitney *U*-test, *χ* ^2^ test, Kaplan–Meier method	Number of cases to reach plateau	OT, console time, continence, C: all show continued improvement over first 50 with no plateauPSM: no improvement over first 50
Good *et al*. (2015)^37^	RALP (transperitoneal), UK	OT, EBL, C, PSM, continence (at 3 months)	Split-group, LOWESS; Mann–Whitney *U* test, Pearson *χ* ^2^ test	Number of cases to reach plateau	OT: 250EBL: 250PSM (overall): 300PSM (pT2): 300PSM (pT3): No plateau reachedC: 250Continence: 100
Gumus *et al*. (2011)^38^	RALP (extraperitoneal), Turkey	OT, EBL, TR, PSM, BCR, LOS, continence, potency	Split-group (thirds); *χ* ^2^ test, ANOVA, Kruskal–Wallis test	Number of cases to achieve comparable outcomes to the published results of high-volume centres	80–120
Hashimoto *et al*. (2013)^39^	RALP, Japan	OT, EBL, PSM, C, continence	Split-group; *χ* ^2^ test, Spearman’s rank correlation test, Mann–Whitney *U*-test	Number of cases to achieve ‘acceptable’ outcomes	OT: 25PSM, C: 50Continence: 100
Herrell and Smith (2005)^40^	RALP, USA	EBL, haematocrit decrease, TR, LOS, post-op pain, QoL, PSM, continence, potency	NR	Number of cases to achieve outcomes comparable to open approach	>150
Islamoglu *et al*. (2018)^41^	RALP (transperitoneal), Turkey	OT, LOS, haematocrit decrease, PSM, BCR	Moving average method, split-group; Fisher’s exact test, Pearson’s *χ* ^2^ test, Student’s *t*-test, Mann–Whitney *U*-test	Number of cases to reach plateau	OT: 50
Ko *et al*. (2009)^42^	RALP (transperitoneal), South Korea	OT, setup time, console time, EBL, LOS, C, TR, PSM, urine leakage on cystogram at 14 days, continence, potency	Split-group; Spearman’s rank correlation test, *χ* ^2^ test, Mann–Whitney *U*-test	Number of cases for TR and urine leakage to reach zero	TR: >15Urine leakage on cystogram at 14 days: >20
Lee *et al*. (2010)^43^	RALP (transperitoneal), South Korea	OT, EBL, PSM, C, LOS	Split-group; ANOVA, *χ* ^2^ test, Spearman’s rank correlation test	Number of cases to reach plateau	OT, EBL: >24PSM: continuous decrease with no plateau
Monnerat Lott *et al*. (2018)^45^	RALP (transperitoneal), Brazil	OT, console time, EBL, PSM, C, continence, potency	Split-group; Fisher’s exact test, *χ* ^2^ test, ANOVA, Tukey test	Number of cases to reach plateau	Continence, potency, PSM: plateau not reached in first 100
Maddox *et al*. (2013)^44^	RALP (extraperitoneal), USA	OT, console time, C, PSM, TR, CR	Split-group (quintiles); ANOVA	Number of cases to reach plateau	Minor C: stable throughoutMajor C: drop after 100
Ohwaki *et al*. (2020)^46^	RALP, Japan	OT, EBL, PSM	CUSUM method; *χ* ^2^ test, Kruskal–Wallis test	Number of cases to achieve competency	PSM: 45
Ou *et al*. (2011)^47^	RALP, Taiwan	OT, console time, EBL, C, TR, PSM	Split-group (quartiles); Mann–Whitney *U*-test, Fisher’s exact test	Number of cases to significantly reduce complications	C, TR: 150
Pardalidis *et al*. (2008)^48^	RALP (transperitoneal), Greece	OT, EBL, C, LOS, pain, continence, potency, PSM	Split-group; no tests for statistical significance reported	NR	10–12
Patel *et al*. (2005)^49^	RALP, USA	OT, EBL, C, LOS, PSM, continence	Split-group (quartiles); no tests for statistical significance reported	Number of cases to achieve outcomes comparable to open approach	20–25
Ploussard *et al*. (2010)^50^	RALP (extraperitoneal), France	OT, EBL, TR, C, CR, LOS, PSM continence, potency	Split-group (quintiles); *χ* ^2^ test, Fisher’s exact test, Mann–Whitney *U*-test	Number of cases to achieve competency	OT: 10 (for 3 h proficiency)EBL, C, TR, LOS: ≈30PSM (pT2): ≈60
Samadi *et al*. (2007)^51^	RALP, USA	OT, EBL, LOS, duration of catheterisation, continence	Split-group (quartiles); tests used for statistical significance were not reported	Number of cases to reach plateau	Plateau not reachedGreatest improvements occurred in first 20
Sammon *et al*. (2010)^52^	RALP (extraperitoneal), USA	OT, EBL, LOS, BNC, PSM	Graphical inspection, non-linear regression model; *t*-tests, Fisher’s exact test	Number of cases to reach plateau	OT: 25
Sharma *et al*. (2011)^53^	RALP (extraperitoneal), UK	OT, EBL, LOS, C, CR, PSM, continence, potency	Moving average, split-group; multivariable logistic regression, *χ* ^2^ test	Number of cases to reach plateau	OT, EBL: continuous improvement, no plateau reached in 330
Sivaraman *et al*. (2017)^54^	RALP, France	PSM, BCR	CUSUM method, logistic regression; Kaplan–Meier curves, Cox regression	Number of cases to reach ‘transition point’ (plateau) in the learning phase	PSM, BCR: 100
Slusarenco *et al*. (2020)^55^	RALP (transperitoneal), Russia	OT, EBL, C, CR, TR, PSM, duration of catheterisation, continence, potency	Split-group; tests used for statistical significance were not reported	Number of cases to reach 3 h proficiencyNumber of cases to achieve median blood loss (150 ml)	OT: >80EBL: 50
Song *et al*. (2020)^56^	RALP (transperitoneal), South Korea	OT, EBL, C, PSM, continence, potency	Graphical inspection with LOWESS; independent *t*-test, Pearson’s *χ* ^2^ test	Number of cases to reach lowest point for each outcome	OT: 200EBL: 230
Thompson *et al*. (2018)^57^	RALP, Australia	PSM, QoL, BCR	Modelled as natural log function; logistic regression, Cox regression	Number of cases to reach plateau	PSM (pT2): 477PSM (pT3/pT4): 360BCR: 226
van der Poel *et al*. (2012)^58^	RALP, Netherlands	OT, time for lymph node dissection, number of removed lymph nodes, % patients with nodal metastases, C	Split-group: univariate and multivariate logistic regression models	Number of cases to reach plateau	OT: 150Lymph node yield: 250Node positivity rate: 300C: 400
Williams *et al*. (2011)^59^	RALP (transperitoneal), Canada	OT, EBL, PSM	CUSUM method; Fisher’s exact test, Kruskal–Wallis test	Number of cases to reach plateau	PSM (pT2): ≈110 (no PSMs in last 50)
Wu *et al*. (2008)^60^	RALP (extraperitoneal), Taiwan	OT, console time, EBL, TR, C, PSM, continence	Graphical inspection, split-group; no tests for statistical significance reported	Number of cases to reach plateau	18
Baumert *et al*. (2004)^61^	LRP (transperitoneal), France	PSM	Split-group (quartiles); *χ* ^2^ test, Fisher’s exact test, Student’s *t*-test, ANOVA	NR	PSM length and rate significantly lower in last 50 cases
Di Gioia *et al*. (2013)^62^	LRP (transperitoneal), Brazil	OT, anastomosis time, EBL, LOS, C, continence, potency, PSM	Split-group (thirds); Kruskal–Wallis test, *χ* ^2^ test, Fisher’s exact test	Number of cases to reach potency	OT, anastomosis time: 80
Dias *et al*. (2017)^63^	LRP (transperitoneal), Brazil	OT, EBL, LOS, C, continence, PSM, CR, TR	Split-group (quartiles); Fisher’s exact test, Kruskal–Wallis test, ANOVA, Tukey test, logistic regression	Number of cases to reach plateau	OT: 40 (plateaued at 150 min)Continence: 70 (plateaued at 95%)
Eden *et al*. (2009)^64^	LRP (transperitoneal initially, then extraperitoneal), UK	OT, EBL, TR, CR, LOS, C, PSM, continence, potency	Logistic regression model; independent samples *t*-test, Fisher’s exact test	Number of cases to reach plateau	OT, EBL: 100–150C, continence: 150–200Potency: 700
Eden *et al*. (2013)^65^	LRP (transperitoneal, ePLND)	OT, EBL, TR, LOS, C (generic and PLND-specific), lymph node yield	CUSUM method; independent samples *t*-test, Fisher’s exact test	Number of cases to reach plateauNumber of cases to achieve acceptable failure rate (FR)	OT: 130 (plateau)C (generic): 136 (10% FR), 346 (5% FR)C (PLND-specific): 40 (5% FR)Lymph node yield: 150 (plateau)
Good *et al*. (2015)^37^	LRP, UK	OT, EBL, C, PSM, continence (at 3 months)	Split-group, LOWESS; Mann–Whitney *U*-test, Pearson *χ* ^2^ test	Number of cases to reach plateau	OT: 250EBL: 250PSM (overall): 200PSM (pT2): 250PSM (pT3): 200C: 250Early continence: 350
Handmer *et al*. (2018)^66^	LRP, Australia	OT, EBL, TR, CR, PSM	Split-group (first 100 vs. second 100); Pearson’s *χ* ^2^ test	NR	Improvements noted in second 100 compared to first 100, no significant improvement in PSM rates between the two groups
Hruza *et al*. (2010)^67^	LRP (transperitoneal initially, then extraperitoneal), Germany	OT, EBL, LOS, PSM, NSM, lymph node status, C	Split-group; Pearson *χ* ^2^ test, Fisher’s exact test, logistic regression model	Number of cases to reach plateau	C: 700 (1st gen.), 250 (3rd gen.)
Mason *et al*. (2016)^68^	LRP (extraperitoneal), UK	OT, EBL, LOS, C, PSM	Split-group; multivariable regression models	Number of cases to reach plateau	LOS: 100PSM (pT2): 150
Mitre *et al*. (2013)^69^	LRP (extraperitoneal), Brazil	OT, EBL, TR, C, CR, PSM, BCR	Split-group (thirds); *χ* ^2^ test, ANOVA, Bonferroni test	Number of cases for significant decreaseNumber of cases to reach plateau	C, CR: significant decrease after first 51OT, EBL, PSM: 110
Poulakis *et al*. (2005)^70^	LRP (transperitoneal initially, then extraperitoneal), Germany	OT, EBL, LOS, PSM, BCR, difficulty scores (relative to open RP), duration of catheterisation	Multivariate regression; *t*-test, Fisher’s exact test, linear regression	Number of cases to achieve rapid decrease in outcome	OT: 21
Rodriguez *et al*. (2010)^71^	LRP (extraperitoneal), USA	OT, EBL, PSM, LOS, TR	Split-group (quartiles); Kruskal–Wallis test, Fisher’s exact test, *χ* ^2^ test, Wilcoxon rank-sum test	Numbers of cases for significant decrease	OT: 100PSM (pT2): 200
Secin *et al*. (2010)^72^	LRP, international	PSM	Split-group; multivariable regression models	Number of cases to reach plateau	PSM: 200–250
Sivaraman *et al*. (2017)^54^	LRP, France	PSM, BCR	CUSUM method, logistic regression; Kaplan–Meier curves, Cox regression	Number of cases to reach ‘transition point’ (plateau) in the learning phase	PSM, BCR: 350
So *et al*. (2011)^73^	LRP (mostly transperitoneal, last 11 were extraperitoneal), South Korea	OT, EBL, PSM, continence, potency	Split-group; Student’s *t*-test, *χ* ^2^ test, Fisher’s exact test, Mann–Whitney *U*-test, Kruskal–Wallis test, Spearman’s rank correlation test, logistic and linear regression models	Number of cases to reach plateau	OT: 40
Vickers *et al*. (2009)^74^	LRP, European and North American institutions	BCR	Linear and logistic regression models; multivariable, parametric survival-time regression model	NR	No plateau, absolute risk difference of ~10% for BCR at 5 years between the most and least experienced surgeons
Bajalia *et al*. (2020)^75^	RAPN, USA	OT, Trifecta (NSM, WIT, C), functional volume loss	Multivariable logistic and linear regression models; 2-sided statistical tests	Number of cases to reach plateauNumber of cases to maximise outcomes	Trifecta: ≈77OT: 77
Castilho *et al*. (2020)^76^	RAPN, Brazil	OT, EBL, Trifecta (NSM, WIT, C)	Split-group (halves); Mann–Whitney *U*-test, Friedman paired test, *χ* ^2^ test, Fisher’s exact test, logistic regression	Number of cases to achieve >60% Trifecta rate	Trifecta: 50
Dias *et al*. (2018)^77^	RAPN, India	OT, console time, LOS, EBL, Trifecta (NSM, WIT, C)	Split-group; ANOVA, Pearson’s correlation test, Spearman’s rank correlation test	Number of cases to achieve OT <120 min, WIT <20 min, EBL <100 ml	OT: 44WIT: 44EBL: 54
Hanzly *et al*. (2015)^78^	RAPN, USA	OT, WIT, EBL, post-op eGFR, C	Split-group (quartiles); Wilcoxon rank-sum test, Fisher’s exact test	Number of cases to achieve mastery	OT: gradual declineC: undefined learning curveWIT: learning curve reached between cases 29 and 58
Haseebuddin *et al*. (2010)^79^	RAPN, USA	OT, WIT	Polynomial regression; Student’s *t*-test	Numbers of cases to reach plateau	OT: 16WIT: 26
Larcher *et al*. (2019)^80^	RAPN, 2 European centres	WIT, C, PSM	Multivariable logistic and linear regression models; 2-sided statistical tests	Number of cases to reach plateau	WIT: 150C: no plateau reached in first 300
Lavery *et al*. (2011)^81^	RAPN, USA	OT, WIT, EBL, LOS, C	Graphical inspection; *χ* ^2^ test, Student’s *t*-test	Number of cases to match or better the average OT and WIT of the last 18 LPNs performed by the surgeon (179.7 and 24.7 min, respectively)	OT: 5WIT: 5
Motoyama *et al*. (2020)^82^	RAPN, Japan	OT, console time, EBL, Trifecta (NSM, WIT, C), LOS, TR	Split-group (quintiles); ANOVA, logistic regression	Number of cases to achieve console time ≤150 min, WIT ≤20 min	Console time: 6WIT: 4
Mottrie *et al*. (2010)^83^	RAPN, Belgium	Console time, WIT, EBL, pelvicalyceal repair, C, PSM	Split-group; ANOVA, Kruskal–Wallis test, Pearson *χ* ^2^ test, paired-samples *t*-test	Number of cases to achieve console time <100 min, WIT <20 min	Console time: 20WIT: 30
Omidele *et al*. (2018)^84^	RAPN, USA	OT, EBL, LOS, Trifecta (NSM, WIT, C), decrease in post-op eGFR	Split-group (quartiles); paired-samples *t*-test	Number of cases to achieve:- 0 C- NSM- WIT ≤25 min- Decrease in post-op eGFR ≤15%	>61–90
Pierorazio *et al*. (2011)^85^	RAPN, USA	OT, WIT, EBL	Split-group; *t*-test, *χ* ^2^ test, ANOVA	Number of cases to reach plateau	No significant learning curve identified
Tobis *et al*. (2012)^86^	RAPN, USA	OT, EBL, WIT, CR, LOS, PSM	Split-group (halves); Mann–Whitney *U*-test, Fisher’s exact test, multivariable linear regression models	Number of cases to reach plateau	No plateaus reachedOT decreased as surgeon experience increased
Xie *et al*. (2016)^87^	RAPN, China	OT, EBL, MIC (NSM, WIT, C)	Split-group (quartiles); Mann–Whitney *U*-test, ANOVA, Kruskal–Wallis test, Pearson’s *χ* ^2^ test	Number of cases to achieve MIC rate >80%	MIC: ≈75
Zeuschner *et al*. (2021)^88^	RAPN, Germany	OT, EBL, Trifecta (NSM, WIT, C), MIC, CR, LOS	Logistic and linear regression analysis; Spearman’s rank correlation test, Fisher’s exact test, Mann–Whitney *U*-test, ROC analysis	Number of cases to become ‘experienced’ (have a 70% probability of achieving MIC or Trifecta)	>35
Azawi *et al*. (2014)^89^	HALPN (with early arterial clamp removal), Denmark	OT, MIC (NSM, WIT, C), LOS, LKF	Split-group (thirds); Paired *t*-test, Kruskal–Wallis test, multiple regression analysis	Number of cases to achieve 95% MIC rateNumber of cases to achieve WIT ≤5 min	MIC: 40WIT: 40
Bhayani (2008)^90^	LPN, USA	OT, EBL, WIT, C, CR, pelvicalyceal system repair, LOS	Split-group (halves); *t*-test	NR	No significant differences between groups except for LOS
Gaston *et al*. (2004)^19^	HALN, USA	OT, EBL, LOS, difficulty scores	Graphical inspection, split-group; ANOVA	NR	OT, difficulty scores; significantly decreased by case 4EBL, LOS: stable throughout
Gill *et al*. (2001)^91^	LRN, USA	OT, EBL, C, CR, LOS, PSM	Split-group (halves); Spearman’s rank correlation test, Wilcoxon rank-sum test, *χ* ^2^ test, multivariate regression	NR	OT: significant decrease from first 50 to second 50
Gozen *et al*. (2017)^21^	LRN, Germany	OT, EBL, C, TR, PSM, NSM	Split-group; Pearson’s *χ* ^2^ test	Number of cases to reach plateau	C: 40 (1st gen), 25 (3rd gen)
Hanzly *et al*. (2015)^78^	LPN, USA	OT, WIT, EBL, post-op eGFR, C	Split-group (quartiles); Wilcoxon rank-sum test, Fisher’s exact test	Number of cases to achieve mastery	OT, C: undefined learning curveWIT: learning curve reached between cases 58 and 87
Jeon *et al*. (2009)^92^	LRN, South Korea	EBL, C, TR	Split-group; independent *t*-test, Pearson’s *χ* ^2^ test, Fisher’s exact test, ANOVA, Dunnett *t*-tests	Number of cases to achieve competence	15
Kanno *et al*. (2006)^93^	LN, Japan	OT, C	Split-group; Student’s *t*-test, *χ* ^2^ test	Number of cases to achieve acceptable outcomes comparable to the published literature	OT, C: 50
Kawauchi *et al*. (2005)^94^	HALN, Japan	OT, EBL, C, CR	Split-group; Student’s *t*-test	Number of cases to gain ‘average’ operating skills	OT: 5–10
Link *et al*. (2005)^95^	LPN, USA	OT, WIT	Stepwise linear regression model	Association between surgeon experience and outcome	OT: significant association with surgeon experience
Masoud *et al*. (2020)^96^	LN, Egypt	OT, EBL, TR, CR, LOS, C	Split-group (halves); Fisher’s exact test, Student’s *t*-test, Mann–Whitney *U*-test	Number of cases to reach plateau	OT:22
Pierorazio *et al*. (2011)^85^	LPN, USA	OT, WIT, EBL	Split-group; *t*-test, *χ* ^2^ test, ANOVA	Number of cases to reach plateau	25
Porpiglia *et al*. (2013)^97^	LPN, Italy	MIC (NSM, WIT, C)	Split-group (quartiles); Mann–Whitney *U*-test, Kruskal–Wallis test, ANOVA	Number of cases to achieve acceptable MIC rate (80%)	150
Collins *et al*. (2014)^98^	RARC (with intracorporeal neobladder), Sweden	OT, EBL, C, CR, lymph node yield, PSM, LOS	Split-group; Jonckheere–Terpstra test, ANOVA, Mann–Whitney *U*-test, Fisher’s exact test, Brown–Forsythe test	Decrease in outcomes with increasing surgeon experience	OT, C, CR, LOS: decreases noted with increasing experienceEBL, PSM, LOS: stable throughout
Dell’Oglio *et al*. (2020)^99^	RARC (with intracorporeal urinary diversion), Belgium	OT, lymph node yield, PSM, C, 18-month recurrence	Multivariable linear and logistic regression models, LOWESS; tests used for statistical significance were not reported	Number of cases to reach plateau	OT: 50
Guru *et al*. (2009)^100^	RARC (with ePLND), USA	OT, cystectomy time, PLND time, EBL, PSM, C, LOS, lymph node yield	Graphical inspection, split-group (quartiles); logistic regression model	Number of cases to reach plateau (where a <1% change occurred in the outcome)	OT: 16EBL: 11LOS:12Lymph node yield: 30
Hayn *et al*. (2011)^101^	RARC, USA	OT, EBL, C, PSM, lymph node yield	Split-group (thirds); *χ* ^2^ test, Kaplan–Meier survival analyses	Association between sequential case number and outcome	OT, lymph node yield: significant association with surgeon experienceC, EBL, PSM: no significant association
Hellenthal *et al*. (2010)^102^	RARC, 15 international centres	PSM	Split-group; logistic regression model	Association between sequential case number and outcome	PSM: no significant association
Honore *et al*. (2019)^103^	RARC, Australia	OT, EBL, LOS, C, PSM, lymph node yield	Split-group (halves); Student’s *t*-test, Mann–Whitney *U*-test	Decrease in outcomes with increasing surgeon experience	OT: significant reduction from first 50 to second 50
Pruthi *et al*. (2008)^104^	RARC (with extracorporeal urinary diversion, USA	OT, EBL, C, PSM, lymph node yield, bladder entry, time to flatus, time to bowel movement, LOS	Graphical inspection, split-group (quintiles); multiple paired regression models	Number of cases to reach plateau	OT, EBL: 20
Wang *et al*. (2019)^105^	RARC (with intracorporeal urinary diversion), USA	OT, lymph node yield, ureteral stricture rate, PSM	CUSUM method; no tests for statistical significance reported	Number of cases to reach plateau	OT: 10–11Lymph node yield, ureteral stricture rate, PSM: no learning curve noted
Aboumarzouk *et al*. (2012)^106^	Laparoscopic radical cystectomy (with PLND and urinary diversion), Poland	OT, EBL, lymph node yield, LOS, morphine requirement, C	Split-group; Mantel–Haenszel *χ* ^2^ test, inverse variance analysis	NR	OT: significantly decreased with increasing surgeon experience
Chammas *et al*. (2019)^107^	Robotic pyeloplasty (adult), Brazil	OT, suturing time, LOS, EBL, C, CR	Split-group (quartiles); Kruskal–Wallis test, ANOVA	Number of cases to achieve significant decrease in outcomes	OT, LOS: 25
Cundy *et al*. (2015)^108^	Robotic pyeloplasty (paediatric), UK	OT, individual step time, C, CR	CUSUM method; Student’s *t*-test, ANOVA, Kruskal–Wallis test	Number of cases of cases to transition beyond learning phase	OT: 57Setup time: 10Docking time: 15Console time: 42
Dothan *et al*. (2021)^109^	Robotic pyeloplasty (paediatric), Israel	OT, LOS, C	Split-group (early vs. late phase); Student’s *t*-test, Mann–Whitney *U*-test, *χ* ^2^ test, Fisher’s exact test	Significant decrease in outcome between early and late phase	No significant decrease in any outcome with increasing surgeon experience; short learning curve
Kassite *et al*. (2018)^110^	Robotic pyeloplasty (paediatric), France	OT, adjusted OT (AOT), composite outcome	CUSUM method; Student’s *t*-test, Mann–Whitney *U*-test	Number of cases to achieve proficiency	OT: 23AOT: 19Composite outcome: 22
Sorensen *et al*. (2011)^111^	Robotic pyeloplasty (paediatric), USA	OT, post-op pain, LOS, EBL, C	Logistic regression model; *χ* ^2^ test, Student’s *t*-test	Number of cases to achieve ‘rudimentary’ proficiency (achieve comparable outcomes to those of open surgery)	15–20
Tasian *et al*. (2013)^112^	Robotic pyeloplasty (paediatric), USA	Console time	Linear regression model; Fisher’s exact test, Kruskal–Wallis test, Mann–Whitney *U*-test	Number of cases for fellows to achieve comparable outcomes to those of the attending	Console time: 37 (projected)
Calvert *et al*. (2008)^113^	Laparoscopic pyeloplasty (adult), UK	OT, LOS, time to normal diet, Hb drop, C, CR	Split-group; Student’s *t*-test, *χ* ^2^ test	Number of cases for significant decrease	C, CR: ≈30
Panek *et al*. (2020)^114^	Laparoscopic pyeloplasty (paediatric), Poland	OT, LOS, failure rate (failure being any surgical reintervention at the ureteropelvic junction)	Split-group; Fisher’s exact test	NR	Failure rate: no change, comparable to previous studies
Janetschek *et al*. (2000)^22^	Laparoscopic RPLND, Austria	OT, C	Split-group; no tests for statistical significance reported	Number of cases to achieve outcomes comparable to open surgery	OT: continued reduction over first 64
Schermerhorn *et al*. (2021)^23^	Robotic RPLND, USA	OT, setup time, lymph node count, C	Linear and logistic regression models; tests used for statistical significance not reported	Predicted number of cases to decrease OT by 1 h	OT: 44 (predicted)

ANOVA, analysis of variance; BCR, biochemical recurrence; BNC, bladder neck contractures; C, complications; CR, conversion rate; CUSUM, cumulative sum; EBL, estimated blood loss; eGFR, estimated glomerular filtration rate; EPIC, Expanded Prostate Cancer Index Composite; ePLND, extended pelvic lymph node dissection; exp., experienced; gen., generation; HALN, hand-assisted laparoscopic nephrectomy; HALPN, hand-assisted laparoscopic partial nephrectomy; Hb, haemoglobin; LKF, loss of kidney function; LN, lymph nodes; LOS, length of stay; LOWESS, Locally Weighted Scatterplot Smoothing; LPN, laparoscopic partial nephrectomy; LRN, laparoscopic radical nephrectomy; LRP, laparoscopic radical prostatectomy; MIC, ‘Margins, Ischaemia, and Complications’ score; NR, not reported; NSM, negative surgical margins; OT, operative time; PLND, pelvic lymph node dissection; PSM, positive surgical margins; QoL, quality of life; RALP, robot-assisted laparoscopic prostatectomy; RAPN, robot-assisted partial nephrectomy; RARC, robot-assisted radical cystectomy; ROC, receiver operating characteristic; RP, radical prostatectomy; RPLND, retroperitoneal lymph node dissection; TR, transfusion rate; WIT, warm ischaemia time.

#### Robot-assisted laparoscopic prostatectomy (RALP)

Thirty-nine studies^18,20,24–60^ evaluated the learning curve of RALP. While the majority of RALP studies focussed on defining the learning curve for OT and positive surgical margins (PSMs), Alemozaffar *et al*.’s study^25^ was unique in defining the learning curve for potency, demonstrating that surgeon experience correlated with improved sexual function at 5 months (*P*=0.007) and 12 months (*P*=0.061) up to a plateau phase of 250–300 nerve-sparing RALP cases. Fossatti *et al*.^35^ reported urinary continence recovery increasing from 60% initially to 90% after 400 procedures, with surgeon experience being a significant predictor of continence recovery (*P*<0.001).

Samadi *et al.* ^51^ evaluated a single surgeon’s first 70 RALPs, noting a sustained downward trend in OT (*P*<0.0001), length of stay (*P*=0.003) and EBL (*P*<0.00001). Gumus *et al.* ^38^ also observed a continued decrease in OT, with it decreasing from 182 min in the first 40 patients to 168 min in the second 40 patients and then down to 139 min in the third 40 patients.

Bravi *et al.* ^28^ found that the risk of PSMs decreased from 15.3% for a surgeon with 10 prior RALPs experience to 6.7% for a surgeon with experience of 250 RALPs. Williams *et al.* ^59^ used the CUSUM method to define the PSM learning curve in RALP, setting acceptable and unacceptable positive margin rates at 10 and 15%, respectively. They concluded that around 110 cases are required to overcome the learning curve for pT2 PSMs.

Van der Poel *et al.* ^58^ reported an increase in lymph node yield and in the node positivity rate, which significantly increased from 4 to 23.1% from the first 50 cases to their 351st–400th cases. A decrease in Clavien–Dindo grade I and II complications were also noted as surgeon experience increased, but this downward trend was not observed for grade III and IV complications.

The four studies^20,48,49,60^ reporting the lowest number of cases to overcome the learning curve notably used carefully selected patients so as to ease the transition for open surgeons to the robotic interface. For example, Pardalidis *et al.* ^48^ initially only included patients with a prostate volume less than 50 cm^3^, Gleason score ≤7, BMI <30 and with no previous major pelvic surgery.

#### Laparoscopic radical prostatectomy (LRP)

Sixteen studies^37,54,61–74^ analysed the learning curve of LRP. Handmer *et al.* ^66^ retrospectively reviewed data from nine Australian surgeons, reporting lower rates of mean blood loss (413 vs. 378 ml), blood transfusions (2.4 vs. 0.8%) and decreased length of stay (2.7 vs. 2.4 days) in the surgeons’ first 100 combined cases compared to the second 100. Di Gioia *et al.* ^62^, examining the first 240 LRPs performed by a surgeon with open and laparoscopic experience, reported a significant decrease in the mean anastomosis time (*P*<0.001) as case number increased, concluding that up to 80 cases were required to achieve a plateau in time-related outcomes.

Mitre *et al*.^69^ noted a significant decrease in intraoperative complications after the first 51 cases (*P*<0.05) alongside a significant decrease in the PSM rates from 29.1 to 21.8 to 5.5% for a single surgeon’s first, second and third groups of 55 patients, respectively. Vickers *et al.* ^74^ reported that surgeons who experienced an open radical prostatectomy achieved significantly poorer results for biochemical recurrence than those naïve to the open procedure (risk difference of 12.3%; 95% CI: 8.8–15.7%). A similar trend was observed with regard to complications by Hruza *et al.* ^67^ , who concluded that 700 cases were required for surgeons experienced in the open approach to overcome the learning curve compared to 250 cases for surgeons with minimal open surgical experience.

**Figure 2:**
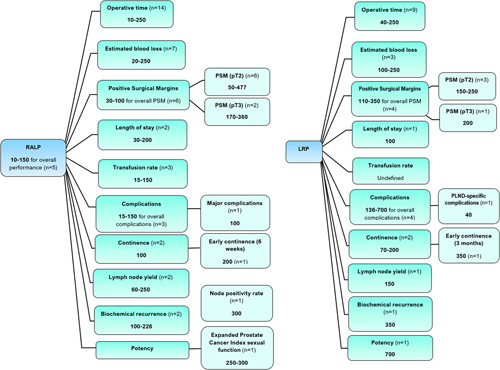
The number of cases required to overcome the learning curve for RALP and LRP. Values are given as a range, with ‘*n*’ denoting the number of studies which numerically defined the learning curve for the outcome. LRP, laparoscopic radical prostatectomy; PLND, pelvic lymph node dissection; PSM, positive surgical margins; RALP, robot-assisted laparoscopic prostatectomy. Where studies defined learning curve for ‘overall performance’, this referred to the number of cases required to achieve competency across the range of outcomes they measured.

Figure 2 summarises the RALP and LRP learning curves.

#### Robot-assisted partial nephrectomy (RAPN)

Fourteen studies^75–88^ analysed the learning curve for RAPN. Mottrie *et al*.^83^ reported a short RAPN learning curve for an experienced robotic surgeon (prior experience of 100 RALPs) with only 20 cases required to achieve a console time of under 100 min. Motoyama *et al*.^82^ reported similar findings with 6 and 4 cases required for a surgeon with prior experience of over 300 RALPs to achieve a console time of under 150 min and a warm ischaemia time (WIT) of under 20 min, respectively.

Bajalia *et al*.^75^ evaluated 418 consecutive RAPNs, concluding that OT decreased by 2.5% per 50% increase in the case number (*P*<0.001) up to the plateau phase at 77 cases. Larcher *et al*.^80^ adjusted for case mix through the use of multivariable regression analyses, reporting that surgeon experience was significantly associated with decreased WIT (*P*<0.0001) and increased probability of a postoperative course without a Clavien–Dindo grade II or higher complication (*P*=0.001). Haseebuddin *et al*.^79^ concluded that the RAPN learning curve for WIT is short at just 26 cases for a surgeon with substantial experience in laparoscopic partial nephrectomy.

#### Laparoscopic partial nephrectomy

Five studies^78,85,90,95,97^ evaluated the learning curve for laparoscopic partial nephrectomy. Bhayani *et al*.^90^ evaluated the first 50 laparoscopic partial nephrectomy cases of a single surgeon, noting that only the length of hospital stay significantly decreased from 3.1 days in the first 25 patients down to 2.5 days in the last 25 patients (*P*=0.01). Porpiglia *et al*.^97^ assessed the laparoscopic partial nephrectomy learning curve using the ‘margin, ischaemia and complication’ (MIC) scoring system, reporting that MIC rates increased from 29.4% in the first 51 patients up to 84.9% in the 150th–206th cases.

#### Other forms of laparoscopic nephrectomy

Three studies^21,91,92^ evaluated the learning curve for laparoscopic radical nephrectomy, while two studies^93,95^ grouped multiple techniques together to map an overall learning curve under the umbrella term of ‘laparoscopic nephrectomy’. Gill *et al*.^91^ analysed a single surgeon’s initial 100 laparoscopic radical nephrectomies, with the only significant decrease between the first 50 and second 50 cases being shorter OT (*P*=0.02).

Three studies^19,89,94^ assessed the learning curve for hand-assisted laparoscopic nephrectomy. Azawi *et al*.^89^ reported achievement of 5 min or less WIT after 40 procedures due to the safe and easily learned procedural step of early arterial clamp removal.

**Figure 3:**
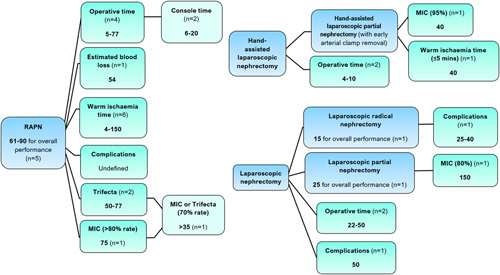
The number of cases required to overcome the learning curve for robot-assisted and laparoscopic nephrectomies. MIC, ‘Margins, Ischaemia and Complications’, score; RAPN, robot-assisted partial nephrectomy. Where studies defined learning curve for ‘overall performance’, this referred to the number of cases required to achieve competency across the range of outcomes they measured.

Figure 3 provides a summary of the RAPN and laparoscopic nephrectomy learning curves.

#### Robot-assisted radical cystectomy

Eight studies^98–105^ analysed the learning curve for RARC. Dell’Oglio *et al*.^99^ used multivariable regression models to adjust for case mix and found that surgeon experience correlated with shorter OT (*P*=0.003), lower 18-month recurrence rates (*P*=0.002) and decreased likelihood of Clavien–Dindo grade II or higher complications 30 days postoperatively (*P*=0.01). Hellenthal *et al*.^102^ found no significant association between sequential case number and PSMs, whereas Guru *et al*.^100^ reported increased lymph node yield from 14 to 23 and decreased PSMs from 4 to 0 between the first 25 cases and 76th–100th cases.

**Figure 4:**
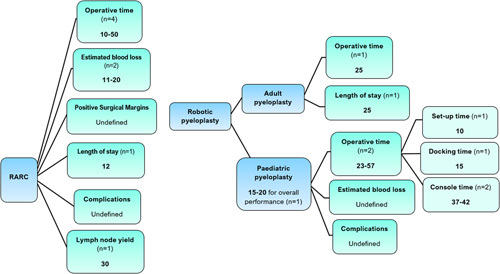
The number of cases required to overcome the learning curve for robot-assisted robotic cystectomy and robotic pyeloplasty. RARC, robot-assisted radical cystectomy. Where studies defined learning curve for ‘overall performance’, this referred to the number of cases required to achieve competency across the range of outcomes they measured.

Figure 4 illustrates a summary of the RARC learning curve.

#### Laparoscopic radical cystectomy

One study^106^ evaluated the learning curve for LRC. Aboumarzouk *et al*.^106^ split a single surgeon’s first 65 LRCs into halves, reporting a significant decrease only in OT (303±28 vs. 285±22.93 min, *P*=0.002), with a nonsignificant decrease in EBL. The learning curve for LRC and the number of cases required to achieve the plateau phase still remain numerically undefined.

#### Robotic pyeloplasty

Six studies^107–112^ evaluated the learning curve for robotic pyeloplasty, one of which involved an adult patient population^107^ whilst the other five involved paediatric patient populations. Chammas *et al*.^107^ analysed 100 consecutive adult robotic pyeloplasty procedures, reporting significant decreases in OT (144.6 vs. 94.6 min, *P*<0.001) and length of stay (7.08 vs. 4.20 days, *P*<0.001) from the first 25 cases to the last 25. Sorensen *et al*.^111^ concluded that only 15–20 paediatric robotic pyeloplasty cases were required to attain an OT with no significant difference to that of open pyeloplasty (*P*=0.23). Cundy *et al*.^108^ identified a classically shaped learning curve for the console time CUSUM chart, but the CUSUM charts for setup and docking times unexpectedly displayed second transition points, which reflected a relocation of the surgical service to a different institution and a change of staff.

The learning curve for robotic pyeloplasty is summarised in Figure 4.

#### Laparoscopic pyeloplasty

Two studies^113,114^ evaluated the learning curve for laparoscopic pyeloplasty.

Calvert *et al*.^113^ retrospectively evaluated the adult laparoscopic pyeloplasty learning curve over 49 procedures. A nonsignificant decrease in conversion rate to open was observed from 18% in the first third of cases to 6% in the last third of cases (*P*=0.53). The authors reported around 30 cases are required to overcome the learning curves associated with complications and conversion rate.

Panek *et al*.^114^ examined the learning curve of paediatric laparoscopic pyeloplasty by splitting a single surgeon’s 95 cases into a group of the first 37 and a group of the remaining 58. A statistically nonsignificant decrease in failure rate (rate of surgical reintervention at the ureteropelvic junction) between the two groups was observed from 16.2 to 5.1% (*P*=0.147).

#### Retroperitoneal lymph node dissection

One study^22^ assessed the learning curve for laparoscopic RPLND, while another^23^ studied the learning curve for robotic RPLND. Janetschek *et al*.^22^ studied 64 laparoscopic RPLND procedures, noting a trend for decreased mean OT from 480 min in the first 14 patients to 222 min in the last 19, thus suggesting the presence of a learning curve. Schermerhorn *et al*.^23^ used linear and logistic regression models to define the learning curve for robotic RPLND. As case number increased, OT and overall complications decreased (*P*=0.001 and *P*=0.001, respectively), with OT predicted to decrease by 1 h after 44 cases. However, an insufficient number of cases were studied for a plateau phase to be reached. Therefore, the learning curves for both laparoscopic and robotic RPLND remain undefined.

#### Comparative studies

The four studies^37,54,78,85^ examining the learning curves of both laparoscopic and robotic procedures also undertook comparative analyses of these. Good *et al*.^37^ identified similar, lengthy learning curves for RALP and LRP but noted that RALP carries the advantages of comparatively lower PSM rates and improved early continence rates. With regard to PSM and biochemical recurrence rates, Sivaraman *et al*.^54^ identified a shorter learning curve of 100 cases for RALP compared to 350 cases for LRP.

Hanzly *et al*.^78^ reported that RAPN has a shorter learning curve for OT than LPN and more effectively preserves the estimated glomerular filtration rate (eGFR) postoperatively. Furthermore, the authors identified that the learning curve for WIT was reached between cases 29 and 58 for RAPN and between cases 58 and 87 for LPN. Pierororazio *et al*.^85^ similarly reported superior outcomes in OT, WIT and EBL for the RAPN cohort compared to the LPN cohort.

## Discussion

### Summary

This systematic review evaluated the existing evidence base for the learning curves of major robotic and laparoscopic urological procedures. For all procedures, the learning curve values varied substantially depending on the outcome measure used to define it, differing learning curve definitions and also on the surgeons’ prior surgical experience. Prior surgical experience was not consistently reported and was poorly quantified with general labels of ‘experienced in open and/or laparoscopic surgery’^20,27,34^, providing no precise indication of the number of procedures performed. Multiple studies report that previous laparoscopic experience does reduce the robotic surgery learning curve in the clinical setting, particularly with regard to OT^115,116^, so it is important for authors to report it in order to contextualise the learning curve value.

OT was the metric most commonly used by the included studies to define the procedural learning curves. However, no uniform definition of OT was used, with one study defining it as the time between the carbon dioxide gas going on and off^73^, whereas another described it as the time between ‘knife-to-skin’ and wound closure^108^. pT3-specific PSM learning curves were defined by several studies^34,37,57^; this is a much more tumour-dependent outcome than surgeon-dependent (whereas the reverse is true for pT2 PSM), so it has limited validity in assessing surgeon performance on the basis of oncological parameters^68^.

Complications and LOS were amongst the patient-outcome variables used to define learning curves, but these also have limitations. LOS is not necessarily representative of the patient’s condition leaving the hospital^64^, given it can be affected by patient wishes and even the day of the week that the procedure is performed due to the varied distribution of hospital resources and ancillary support across the week^117^.

Complications also are not always completely reflective of the surgeon’s performance as they correlate with the quality of complication reporting which thus introduces potential bias to the results^118^. Urinary incontinence is one of the most important patient-outcome variables for RALP and LRP, given its nature as the most disruptive side-effect post-prostatectomy^119^, but its learning curve was only defined by 6 of the 53 included prostatectomy studies.

Learning curve metrics are also subject to the effects of other confounders. In the context of urological robotic surgery, the format of training undergone by the surgeon influences the learning process and hence the rate at which safe surgical outcomes are achieved^120^. The skills and expertise of the whole operative team have also been reported to affect surgical outcomes in urological procedures. Using an expert bedside assistant has been shown to decrease EBL and PSM early in the learning curve for RALP^121^, with the benefits of a skilled bedside assistant including their ability to handle potential emergencies and thereby decrease the risk of complications, while the console surgeon is seated unscrubbed away from the patient^81^. Gumus *et al.* ^38^ proposed a possible learning curve for anaesthetists on the basis that transfusion rates were disproportionately high relative to the EBL for the initial 80 RALP cases and as the decision to transfuse was made by an anaesthetist naïve to robotics and laparoscopy, their inexperience explained this discrepancy. Furthermore, the presence of mentors and the extent of mentorship received by trainees were poorly reported despite it being demonstrated to affect urological learning curves^122^.

The split-group method of analysing the learning curve lacks the sensitivity to define the exact number of cases for which learning curve transitions occur^5^, yet it was the most commonly employed method. Regression techniques were also used by studies, but these may also obscure the identification of key learning curve characteristics such as rate and plateau, given the forced match of the best-fit lines on the data collected^8^.

The conventional view of the surgical learning curve having just one ascent and one plateau phase was challenged in this review by reports of a multiphasic learning curve arising from the tendency of surgeons to take on increasingly complex cases as their confidence improved with experience^94,108^. The case mix effect is well-documented in the literature as a key factor in shaping the learning curve^3,123^, particularly in the post-plateau phase^124^.

### Strengths and limitations

All of the included studies were observational in design, which can introduce confounding and selection bias to results^125^, but measurement of learning curves necessitates such a design as they are based on the observation of changes in variables as surgeon experience increases. Fifty-five studies were single-surgeon in design which limits the generalisability and external validity of their reported learning curves given the vast interpersonal variation between surgeons in terms of technical attributes and prior experience^126^. The lack of adjusting for confounders and variation in outcome measures across the included studies in this review is consistent with the findings of reviews assessing the heterogeneity of the surgical learning curve literature bases^127,128^. Another limitation at the study level was the lack of cost-effectiveness analyses to investigate the association between the length of a surgeon’s learning curve and the economic impact on their institution in terms of training costs.

At review-level, the exclusion of conference abstracts has been identified as a source of publication bias^129^, but the justification for doing so is that the data presented in such abstracts frequently involves preliminary results which do not necessarily provide an accurate representation of the eventual findings upon study completion^130^. Another limitation is that no sensitivity analysis was performed to investigate the effect of including studies at high risk of bias on the conclusions formed.

No date restriction was imposed on studies, so the results of older studies may be confounded by the procedural ‘discovery’ curve in which the surgeon is standardising novel techniques as opposed to learning a standard procedure^12^. However, date restriction would not have been an effective way of eliminating bias from technical changes, given that individual institutions appear to undergo their own procedural discovery curves according to their own local experiences of procedures and outcomes, irrespective of study date. One such example is Sharma *et al*.’s^53^ RALP learning curve study in which visual port placement was altered from the paraumbilical space to the umbilical space in order to reduce an initially high rate of port-site hernias, thereby decreasing complications independent of increasing surgeon experience.

Key strengths of this review include its comprehensive search strategy of multiple databases and the grey literature in order to elicit eligible full-text articles and adherence to PRISMA guidelines), Supplemental Digital Content 1, http://links.lww.com/JS9/A423. This review updates the urological learning curve literature base and is the first to examine the literature pertaining to the learning curves of laparoscopic nephrectomy, LRC, laparoscopic pyeloplasty and both robotic and laparoscopic RPLND.

### Implications for research and clinical practice

As is consistent with the findings of previous reviews^5,11,12,17,126^, standardised reporting of outcomes and performance measures is needed in order to reduce heterogeneity and thereby enable a meta-analysis to be performed to combine learning curve values across studies. Given that surgeons’ background expertise affects their learning curve^131^, surgeons’ baseline characteristics must be reported so as to categorise learning curves by prior experience.

There are ethical concerns raised over surgeons learning on real patients given that more experienced surgeons often attain better outcomes^28^, so increasing surgical experience through simulation-based training is a safer method for reducing the learning curve associated with procedures^132^. Mentorship is another educational intervention that has been demonstrated to reduce the surgical learning curve^133^, with the ‘Leipzig Model’ of supervision in LRP being one such example^134^. In this example, the trainee performs all the steps they are competent at with a mentor acting as a first assistant, then the student becomes the first assistant and observes for the remaining steps to enhance the learning process. Indeed, surgical outcomes in RALP have been shown to improve if the console surgeon has first gained experience as a bedside assistant because this role improves troubleshooting ability and confidence in dealing with more challenging cases^135^.

Although underused in the included studies, CUSUM analysis is a well-described method for not only precisely plotting an individual’s learning curve^8^ but also for auditing purposes in the continuous monitoring of a trainee’s competency as has been demonstrated in the context of cataract surgery^136^ and gynaecology^137^. CUSUM charts enable assessment of a trainee’s performance relative to target values and can alert trainers to out-of-control processes, at which point trainees can be instructed to either stop and undergo further education or conduct their next procedures under observation to ensure that patient safety is not compromised while the learning process restabilises^46^.

The results of the comparative studies^37,54,78,85^ support the view that the robot-assisted approaches to radical prostatectomy and partial nephrectomy have a shorter learning curve than the conventional laparoscopic approaches. Across all outcomes, the laparoscopic modality was not found to confer any advantage over the robot-assisted approach with regard to the learning curve. Thus RALP would be recommended over LRP on the basis of accelerated attainment of competency for the outcomes of PSMs, biochemical recurrence and early continence. In a similar fashion, RAPN would be recommended over LPN owing to its shorter learning curves for OT, WIT and EBL.

Finally, the learning curves for LRC and robotic and laparoscopic RPLND remain undefined, so future studies should evaluate hundreds of consecutive cases to enable the identification of the plateau phase for these procedures and assess the learning curves of multiple surgeons to increase the external validity of their findings.

## Conclusion

This systematic review outlines the range of values for the learning curves of major robotic and laparoscopic urological procedures. As has been described in previous reviews, there was substantial variation in the definitions of outcome measures and performance thresholds, with poor reporting of confounders such as prior surgical experience. Although the majority of studies used the split-group method of analysing the learning curve, the CUSUM method is recommended for more precise characterisation of the key learning curve phases as well as for monitoring the competency of surgeons over time as a form of an appraisal. The use of simulation training, mentorship, and gaining experience as a bedside assistant is recommended in order to reduce the learning curve prior to taking full control as the lead surgeon. Lastly, the results of the comparative studies demonstrate that RALP and RAPN have shorter learning curves for a range of metrics compared to their laparoscopic counterparts.

## Ethical approval

Ethical approval was not required.

## Sources of funding

This research did not receive any grant from funding agencies in the public, commercial, or not-for-profit sectors.

## Author contribution

B.C.: planned the review, performed the search strategies, extracted the data and wrote the manuscript; M.S.A.A.: acted as the second reviewer and contributed to writing the manuscript; A.A.: acted as the third reviewer, aided in planning the review, and edited and reviewed the manuscript; A.K., M.S.K., K.A. and P.D.: contributed to writing the manuscript and providing revisions.

## Conflicts of interest disclosure

Baldev Chahal, Abdullatif Aydin, Mohammad S.A. Amin, Azhar Khan, Muhammad S. Khan and Kamran Ahmed have no conflicts of interest or financial ties to disclose. Prokar Dasgupta declares financial ties as Chief Medical Officer for Proximie Ltd. and Chief Scientific Officer for MysteryVibe Ltd. This research did not receive any specific grant from funding agencies in the public, commercial, or not-for-profit sectors.

## Research registration unique identifying number (UIN)

1. Name of the registry: PROSPERO.
2. Unique identifying number or registration ID: CRD42021251186.
3. Hyperlink to your specific registration (must be publicly accessible and will be checked): https://www.crd.york.ac.uk/prospero/display_record.php?RecordID=25 1186.

## Guarantor

Baldev Chahal and Abdullatif Aydin.

## Data availability statement

Search results and extracted data are available on request.

## Provenance and peer review

Not commissioned, externally peer-reviewed.

## Supplementary Material
