# Supplementary material for: The learning curves of major laparoscopic and robotic procedures in urology: a systematic review
Source: Int J Surg. 2023 May 3;109(7):2037–57. doi: 10.1097/JS9.0000000000000345 (PMC10389344; doi:10.1097/JS9.0000000000000345)
Supplement: Supplementary file 3 [file js9-109-2037-s003.docx]

Appendix

Database search strategy:

| **Database** | **Search Terms** |
| --- | --- |
| PubMed | ((learn*) OR (learning curve[MeSH Terms])) AND ((((((((prostatectom*) OR (cystectom*)) OR (nephrectom*)) OR (pyeloplasty)) OR (retroperitoneal lymph node dissection)) OR (prostatectomy[MeSH Terms])) OR (cystectomy[MeSH Terms])) OR (nephrectomy[MeSH Terms])) NOT(review OR letter OR editorial OR abstract) |
| Embase | (((learn* or learning curve) and (prostatectom* or cystectom* or nephrectom* or pyeloplasty or retroperitoneal lymph node dissection)) not (review or letter or editorial or abstract)) |
| Cochrane Library | (learn* OR learning curve[MeSH Terms]) AND (prostatectom* OR cystectom* OR nephrectom* OR pyeloplasty OR (retroperitoneal lymph node dissection) OR prostatectomy[MeSH Terms] OR cystectomy[MeSH Terms] OR nephrectomy[MeSH Terms]) |

_________________________________________________________________________
